# Effect of Dezocine and Dexmedetomidine as Adjuvants in Ropivacaine for Incision Subcutaneous Infiltration Anesthesia on Incision Healing in Diabetic Rats

**DOI:** 10.1002/brb3.71111

**Published:** 2025-11-30

**Authors:** Lang Yu, Bin Gao, Lingling Sun, Jing Mu, Piaopiao Zhang, Qin Zhang, Huanzhong He, He Liu

**Affiliations:** ^1^ Department of Anaesthesiology & Clinical Research Center For Anaesthesia and Perioperative Medicine & Key Laboratory of Anesthesia and Analgesia Application Technology (Huzhou Central Hospital The Fifth School of Clinical Medicine of Zhejiang Chinese Medical University; Huzhou Central Hospital, The Affiliated Central Hospital of Huzhou University) Huzhou Zhejiang Province People's Republic of China

**Keywords:** adjuvant, diabetic, healing, incision subcutaneous infiltration

## Abstract

**Introduction:**

Surgery can induce insulin resistance (IR) in diabetic patients, and severe IR compromises the body's ability to combat infection and shock, impairs the provision of high metabolic energy required post‐surgery, and delays wound healing. Local infiltration anesthesia is commonly employed for postoperative pain management due to its simplicity, cost‐effectiveness, and efficacy. However, research on optimal anesthetic formulations for incisional subcutaneous infiltration anesthesia that promote wound healing in diabetic patients remains limited. This study aims to investigate the effect of dezocine and dexmedetomidine as adjuvants in ropivacaine incision subcutaneous infiltration anesthesia on incision healing in diabetic rats.

**Methods:**

Six groups, each comprising 18 diabetic rats, were randomly assigned. Following wound suturing, 1 mL of liquid was administered as subcutaneous infiltration anesthesia at the incision site. The compositions of the liquids were as follows: Group A received 0.375% ropivacaine, Group B received 0.375% ropivacaine plus 0.05 mg dezocine, Group C received 0.375% ropivacaine plus 0.005 mg dexmedetomidine, Group D received 0.375% ropivacaine plus 0.05 mg dezocine and 0.005 mg dexmedetomidine, Group E received normal saline plus 0.05 mg dezocine and 0.005 mg dexmedetomidine, and Group M received normal saline. The incision healing process was evaluated on Days 3, 7, and 14 post‐suturing. CD31 and CD68 expression levels in the incision tissues were quantified using mean optical density (MOD), and collagen volume fraction (CVF) was determined via Masson staining.

**Results:**

The findings indicated that on Days 3, 7, and 14, Group D exhibited markedly superior incision healing compared to the other five groups. Conversely, Group M demonstrated inferior incision healing relative to the other groups. Microscopic examination revealed that Group D's enhanced function was attributed to improved tissue structure of incision neovascularization, characterized by larger vascular lumen diameters and more densely packed vascular endothelial cells. On Day 3, Group D showed significantly higher CD31 expression levels compared to the other five groups, while Group M exhibited notably lower CD31 expression. This trend persisted on Days 7 and 14, with Group D maintaining substantially higher CD31 expression. Similarly, on Day 3, Group D displayed significantly higher CD68 expression compared to the other groups, whereas Group M had significantly lower CD68 expression. This pattern was consistent on Days 7 and 14 as well. Additionally, Group D demonstrated significantly greater collagen fiber deposition on Days 3, 7, and 14, resulting in a significantly higher CVF compared to the other five groups. In contrast, Group M exhibited considerably lower CVF and reduced collagen fiber deposition.

**Conclusions:**

We hypothesized that the use of ropivacaine for incision subcutaneous infiltration anesthesia, in conjunction with dezocine and dexmedetomidine, may reduce pain stress and modulate the expression of inflammatory cytokines through the PI3Kγ/Akt signaling pathway and OGF‐OGFr channel. This could lead to a reduction in IR production, inhibition of excessive inflammatory responses while preserving beneficial inflammatory reactions, upregulation of GM‐CSF expression, increased fibroblast proliferation, stimulation of capillary proliferation, collagen fiber deposition, and macrophage aggregation, all of which contribute to improved wound healing.

## Graphical abstract

The adjunctive use of dezocine and dexmedetomidine with ropivacaine in incisional subcutaneous infiltration anesthesia may contribute to improved incision healing in diabetic rats.

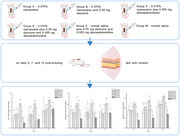

## 1 Introduction

As the third leading cause of death globally and the fourth most significant contributor to years lived with disability, diabetes mellitus represents a critical public health challenge (Umpierrez et al. 2024). In 2021, over 10.5% of the global adult population was diagnosed with diabetes; this proportion is projected to rise to 12.2% (783.2 million individuals) by 2045 (Sun et al. 2022). The annual incidence of perioperative patients with diabetes has been increasing in parallel with the overall prevalence of the disease. Previous research has demonstrated that diabetes adversely affects wound healing following surgery, increases the risk of postoperative infections and other complications (Deng et al. 2021; Yang et al. 2022), and ultimately prolongs hospital stays and escalates healthcare costs (Mieczkowski et al. 2022). Wound repair is a complex, tightly regulated process involving multiple cellular interactions and is essential for maintaining normal skin barrier function (Karnam et al. 2020; Sharifiaghdam et al. 2022). Numerous pathophysiological mechanisms contribute to delayed wound healing in diabetic patients, including excessive activation of the local immune response (Boniakowski et al. 2017; Wolf et al. 2021), oxidative stress (Kunkemoeller and Kyriakides 2017; Modaghegh et al. 2021; Victor et al. 2020), reduced neovascularization, peripheral neuropathy (Burgess et al. 2021; Strand et al. 2024), and accumulation and imbalance in extracellular matrix remodeling (Jin et al. 2025; L. Yan et al. 2024). Unlike typical wound healing processes, diabetic wound healing often deviates from the standard phases, potentially stalling at various stages, particularly during the inflammatory phase. Diabetes impairs immune system function, leading to an accumulation of immune cells at the wound site without completing the immune process or initiating effective repair, resulting in chronic inflammation and delayed wound healing.

Pain has been characterized as a significant factor that can profoundly affect the reciprocal regulation of the neurological, immunological, and endocrine systems; compromise the body's immune response; and influence disease progression (Q. Zhang, Bang, et al. 2022). Most postoperative pain is categorized as inflammatory pain. Following tissue injury, such as surgical incisions, an inflammatory response ensues, leading to the production of compensatory anti‐inflammatory factors and the release of pro‐inflammatory factors. The compensatory anti‐inflammatory factors mitigate injury and inflammation by inhibiting the synthesis of pro‐inflammatory factors. As a critical component of Enhanced Recovery After Surgery (ERAS), subcutaneous infiltration anesthesia at the incision site may alleviate perioperative pain‐related stress, thereby reducing the need for general anesthetics and potentially eliminating the reliance on opioids, which have been widely used in clinical practice. An essential aspect of incision healing involves the accumulation of inflammatory cells at the wound site (Mitsui et al. 2023; Peña and Martin 2024). However, inflammation may reduce the efficacy of local anesthetics (LAs), leading to inadequate analgesia. Current strategies under consideration include the use of adjuvants and increased concentrations of LAs. Therefore, appropriate postoperative analgesia and anesthetic drug formulations can protect the body's beneficial inflammatory response while inhibiting excessive inflammation at the surgical incision, aiding in the recovery of diabetic patients, and meeting ERAS requirements.

According to existing studies, opioid analgesics can induce analgesia in response to tissue damage or inflammation by activating peripheral opioid receptors (Martínez and Abalo 2020). Additionally, opioid analgesics have been shown to enhance collagen synthesis in incision tissues (Mahmoudi and Farahpour 2022). According to reports, the dosage range for subcutaneous injection of dezocine is fairly wide, ranging from 0.4 µg/kg to 10 mg/kg. Furthermore, research has indicated that a lower dosage of dezocine provided via subcutaneous injection can produce the desired effect more successfully (Wu et al. 2021; R. R. Ye et al. 2022; Z. Ye et al. 2018). Our team's previous research results showed that compared with simply using 20 mL of 0.375% ropivacaine for incision local infiltration anesthesia (LIA), for patients undergoing thoracoscopic lobectomy, using 20 mL of 0.375% ropivacaine containing 5 mg dezocine for incision LIA significantly prolonged the duration of the analgesia, reduced the use of systemic opioid drugs, and effectively improved and maintained the inflammatory and anti‐inflammatory responses generated by TNF‐α, IL‐6, and IL‐10 in serum postoperatively. The synergistic effect of opioid agonists or partial agonists on peripheral opioid receptors may contribute to their enhanced analgesic action when combined with LAs. Furthermore, numerous clinical studies have demonstrated that combining LAs with α2 receptor agonists (such as clonidine and dexmedetomidine) or dexamethasone in peripheral nerve blocks can augment analgesic effects and extend their duration (Chen et al. 2023; Desai et al. 2021; Lionel et al. 2019). X. Huang et al. (2017) discovered that administering dexmedetomidine at a dose of 1 or 5 µg around rats' sciatic nerves could alleviate mechanical and thermal pain hypersensitivity, as well as postoperative pain. Moreover, a study has proved that dexmedetomidine added to ropivacaine increased the duration of dense sensory blockade and time for return to normal sensory function in a dose‐dependent fashion (Brummett et al. 2009).

This study aimed to investigate whether subcutaneous incisional infiltration anesthesia using ropivacaine in combination with various adjuvants could enhance collagen synthesis in incision tissues, promote postoperative wound healing, protect the immune system, provide a novel approach for managing postoperative pain in diabetic patients, and introduce a new technique for multimodal postoperative analgesia using a diabetic rat model of incisional injury.

## 2 Materials and Methods

### 2.1 Animals

The Ethics Committee of Huzhou Central Hospital approved this study (Ethics Number: 202112009). This research adhered to the National Institutes of Health (NIH) Guidelines for the Care and Use of Laboratory Animals (2011). A total of 108 male Wistar rats, weighing approximately 100 g, were utilized in this investigation. The animals were housed in a temperature‐controlled environment (22°C–25°C) with a 12‐h light/dark cycle. After 1 week of acclimatization to a standard diet, the rats were fed a high‐sugar and high‐fat diet to induce insulin resistance (IR). In the fourth week, following the high‐sugar and high‐fat diet regimen, the rats received a single intraperitoneal injection of streptozotocin (STZ, 30 mg/kg). One week post‐STZ injection, hyperglycemia was confirmed by measuring blood glucose levels via tail‐vein sampling. Subsequently, diabetic rat models were anesthetized using isoflurane (3%–4% induction, 1.5%–2.5% maintenance, 100% oxygen) to construct a postoperative incision model. Specifically, after complete anesthesia, the dorsal region of the rats was shaved, and the skin was disinfected with povidone‐iodine. A 1 cm transverse incision, 2 mm deep, was made below the dorsal aponeurosis and closed with nonabsorbable sutures. The experimental rats were randomly divided into six groups (*n* = 18 per group). Prior to suturing, subcutaneous infiltration anesthesia was administered with 1 mL of liquid. The compositions of the liquids were as follows: Group A contained 0.375% ropivacaine, Group B contained 0.375% ropivacaine plus 0.05 mg dezocine, Group C contained 0.375% ropivacaine plus 0.005 mg dexmedetomidine, Group D contained 0.375% ropivacaine plus 0.05 mg dezocine and 0.005 mg dexmedetomidine, Group E contained normal saline plus 0.05 mg dezocine and 0.005 mg dexmedetomidine, and Group M contained normal saline. Researchers were blinded to the treatments during the evaluation of experimental outcomes.

### 2.2 Collection of Skin Samples

At 3, 7, and 14 days post‐establishment of the diabetic incision model, six rats from each group were euthanized via intraperitoneal injection of pentobarbital sodium at a dose of 100 mg/kg. A skin sample measuring 1 cm^2^, including the incision site and encompassing the epidermis, dermis, and partial subcutaneous tissue, was excised. The samples were immediately fixed in neutral‐buffered formaldehyde solution (4% formaldehyde) for 24 h.

### 2.3 Preparation of Paraffin Sections

*Dehydration and transparency*: The samples were subjected to dehydration in an incubator at 37°C using a gradient ethanol concentration method. Fixed skin tissues, including incisions, were carefully excised with tweezers and placed on filter paper to facilitate the removal of residual water from high‐concentration ethanol. Specimens underwent sequential dehydration in ethanol concentrations of 60%, 70%, 80%, 90%, 95%, and 100%. The first four concentrations involved 1‐h dehydration cycles, while the latter two concentrations required two separate 30‐min cycles each. To enhance the refractive index of the samples, we performed specimen clarification using a transparent agent following ethanol dehydration. This process involved mixing anhydrous ethanol with xylene in a 1:1 ratio in a sealed container. Specimens were immersed in this mixture for 30 min, followed by immersion in xylene I for 5 min and xylene II for another 5 min. At this stage, the specimens had completed the dehydration and transparency procedures.

*Wax dipping and embedding*: To operate the embedding machine, we first connected the power supply, turned on the switch, set the temperature to between 65°C and 68°C, and activated the freezing function. The paraffin wax was melted in an oven at 60°C, after which the specimen was immersed in the wax thoroughly and evenly for 3 h. A slightly heated embedding device was used to pour a small amount of melted pure paraffin wax (maintained at approximately 60°C) into the skin tissue specimen, ensuring that the transverse section was carefully positioned with tweezers. Subsequently, the specimen was rapidly submerged in pure liquid paraffin. Afterward, the paraffin blocks were cut into square shapes to facilitate slicing with a microtome.

*Slicing and mounting*: The tissue blocks were placed in the microtome for slicing, whereupon 3‐ to 5‐µm‐thick sections were cut and spread on a water bath. These sections were then transferred onto polylysine‐treated anti‐slip slides, numbered, and baked at 65°C for 2 h.

### 2.4 Immunohistochemistry

In this study, rat skin tissues were embedded in paraffin and subsequently processed for immunohistochemistry (IHC). Standard IHC protocols were followed using primary antibodies against CD31 (abcam: ab281583, 1:500) and CD68 (CST: 97778, 1:400). After deparaffinization with xylene, the tissue slides were rehydrated through a graded alcohol series. Following antigen retrieval and blocking, the slides were incubated overnight at 4°C with the respective primary antibodies. The following day, the slides were incubated with secondary antibodies for 1 h at room temperature. Signal detection was performed using an EnVision Detection Kit (Glostrup, Denmark), according to the manufacturer's instructions, with diaminobenzidine (DAB) serving as the chromogenic substrate.

### 2.5 Masson's Trichrome Staining

For Masson's trichrome staining, tissue sections were subjected to sequential staining with hematoxylin, ferric oxide, acid fuchsin, phosphomolybdic acid, and acetic acid. Subsequently, the sections were mounted using neutral gum.

### 2.6 Image Analysis

All specimens were uniformly magnified at a consistent magnification of 20×. The ImageJ image analysis system was utilized to randomly select five fields from each section for analysis. Quantitative assessment of the IHC results was conducted using mean optical density (MOD). Additionally, the collagen fiber content in the tissue surrounding the incision site was quantitatively evaluated and compared via collagen volume fraction (CVF).

### 2.7 Statistical Analysis

Data analysis was conducted using SPSS version 16.0. Results are presented as mean ± SD (standard deviation). For comparisons among multiple groups, one‐way ANOVA was utilized. If homogeneity of variance was confirmed, the LSD test was employed for post hoc pairwise comparisons. In cases where the assumption of homogeneity of variance was violated, Tamhane's T2 test was applied. A *p*‐value less than 0.05 was considered statistically significant.

## 3 Results

**FIGURE 1:**
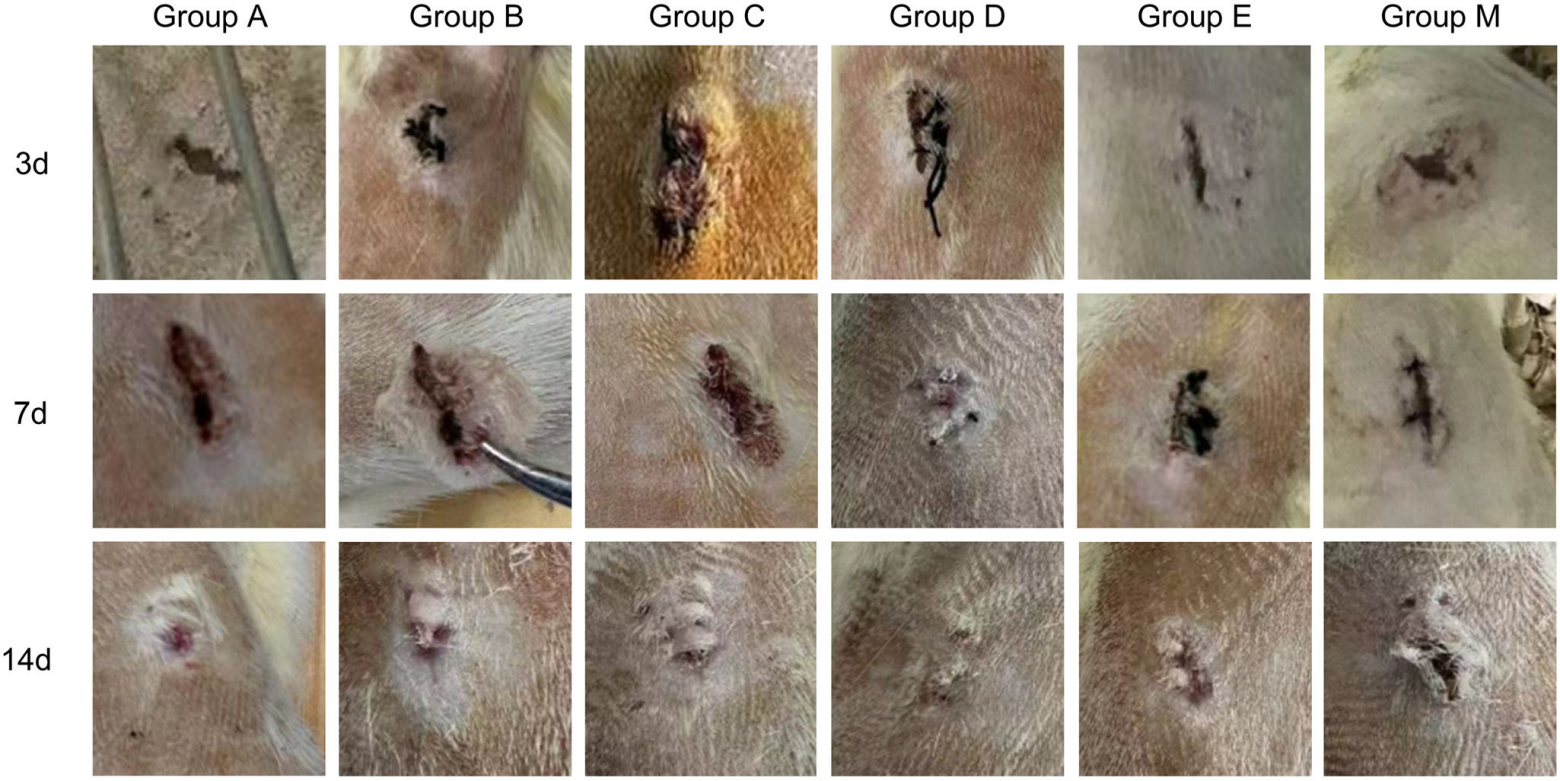
Photographs of the tissue around the incision taken 3, 7, and 14 days following surgery using various anesthetic medication compositions.

We captured images of the incision healing at 3, 7, and 14 days post‐establishment of the diabetic incision model in rats to evaluate the effects of subcutaneous infiltration anesthesia with various adjuvants combined with ropivacaine on wound healing. Our results indicated that, at all observation points (Days 3, 7, and 14), Group D exhibited significantly superior incision healing compared to the other five groups. Conversely, Group M showed markedly inferior healing outcomes relative to the other groups, with the highest incidence of infections and delayed healing. Group D demonstrated the most rapid and infection‐free healing process (Figure 1).

### 3.1 CD31

**FIGURE 2:**
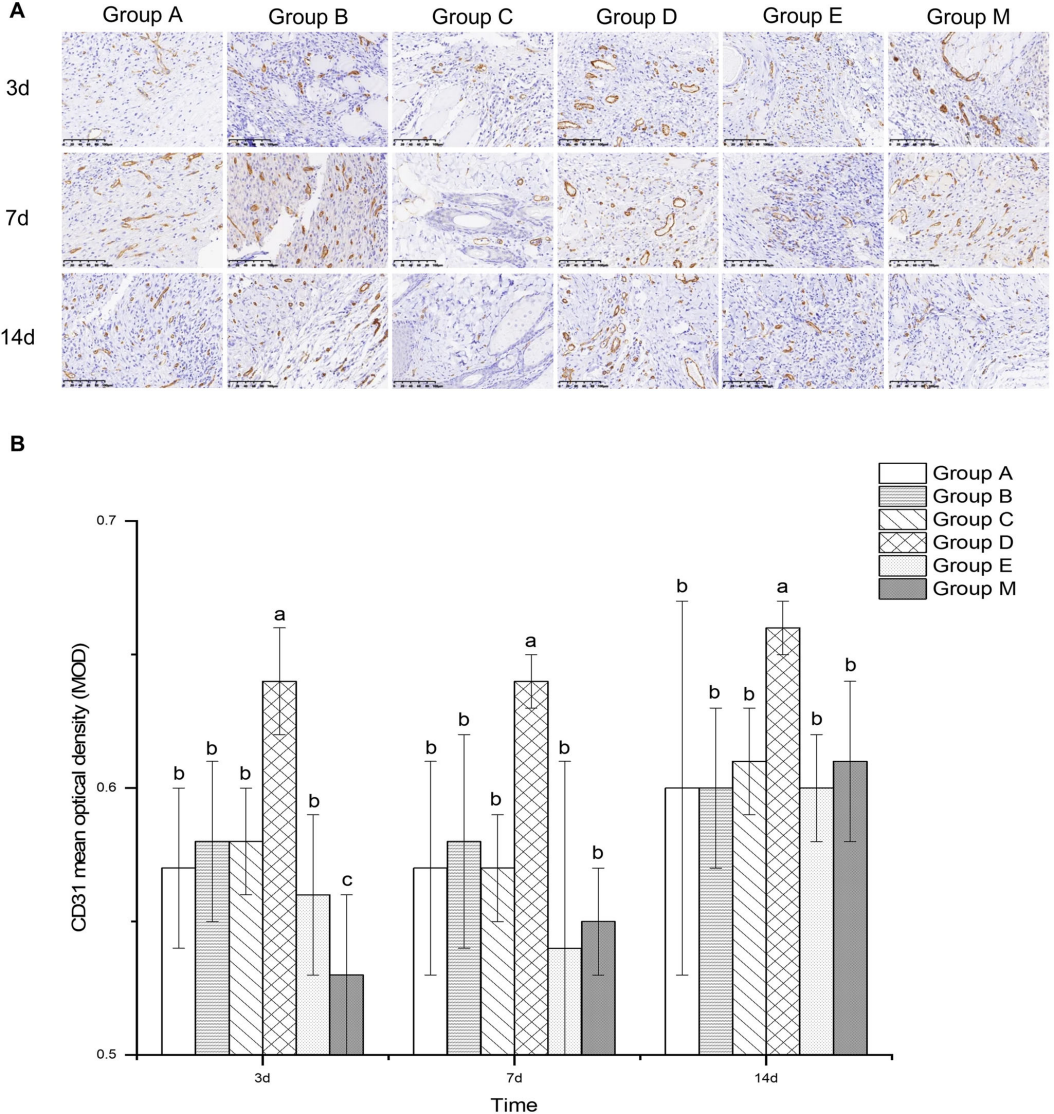
The effects of different anesthetic medication formulations on the expression of CD31 in the incision tissue on 3, 7, and 14 days after surgery. (A) Representative images showing IHC staining for CD31 within incision tissues, at original magnification ×20. (B) Statistical analysis of the expression of CD31 in the incision tissue. Data are expressed as means ± SD (*n*  =  6). For comparisons among multiple groups, one‐way ANOVA was utilized. If homogeneity of variance was confirmed, the LSD test was employed for post hoc pairwise comparisons. In cases where the assumption of homogeneity of variance was violated, Tamhane's T2 test was applied. Letters, such as a, b, and c, on bars indicate clustering on the basis of statistical differences. Values labeled with the same letters are not statistically different from each other, whereas different letters indicate statistical differences (*p* < 0.05).

Granulation tissue formation, which commences 1–2 days post‐wounding, is a critical component of the proliferative phase of incisional wound healing. Neovascularization, characterized by a transient increase in blood vessel density, is an essential aspect of granulation tissue development. In this study, angiogenesis within the incision tissue was evaluated using CD31 immunohistochemical staining at 3, 7, and 14 days following the successful establishment of a postoperative incision injury model in diabetic rats and completion of subcutaneous infiltration anesthesia. Microscopic examination revealed that Group D exhibited a larger vascular lumen diameter and more tightly packed endothelial cells, indicating a robust structural support for neovasculature function (Figure 2A). Statistical analysis of the data showed that on Day 3, CD31 expression was significantly higher in the other five groups compared to Group M (*p *< 0.05), while it was significantly lower in these groups relative to Group D (*p *< 0.05). On Days 7 and 14, Group D demonstrated markedly higher CD31 expression compared to the other five groups, with statistically significant differences (*p *< 0.05). Conversely, no significant differences in CD31 expression were observed among the other groups (Figure 2B). These findings suggest that the use of the Group D drug formulation for subcutaneous infiltration anesthesia may enhance vascular density in incision tissues, thereby promoting granulation tissue formation and wound healing.

### 3.2 CD68

**FIGURE 3:**
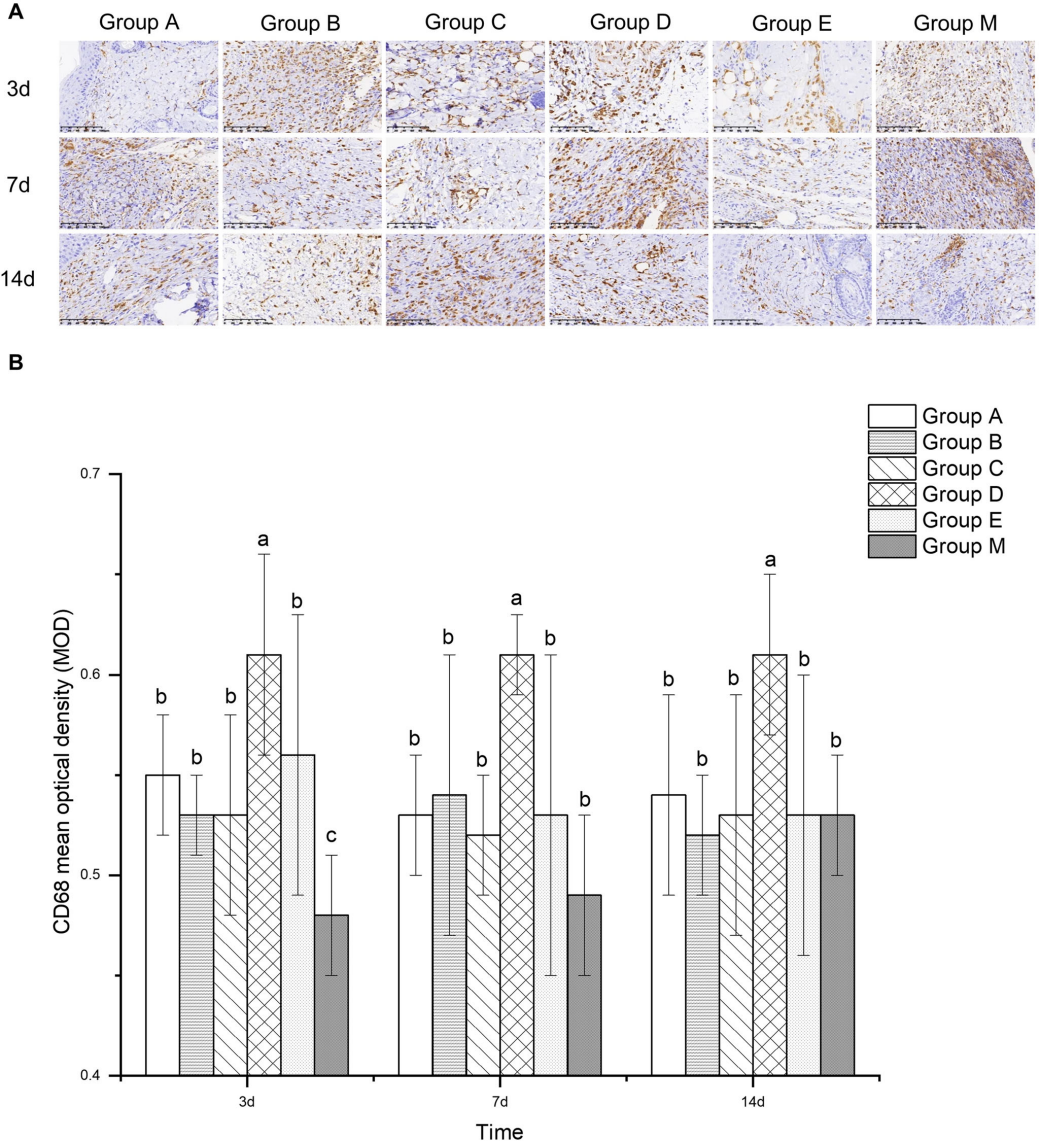
The effects of different anesthetic medication formulations on the expression of CD68 in the incision tissue on 3, 7, and 14 days after surgery. (A) Representative images showing IHC staining for CD68 within incision tissues, at original magnification ×20. (B) Statistical analysis of the expression of CD68 in the incision tissue. Data are expressed as means ± SD (*n*  =  6). For comparisons among multiple groups, one‐way ANOVA was utilized. If homogeneity of variance was confirmed, the LSD test was employed for post hoc pairwise comparisons. In cases where the assumption of homogeneity of variance was violated, Tamhane's T2 test was applied. Letters, such as a, b, and c, on bars indicate clustering on the basis of statistical differences. Values labeled with the same letters are not statistically different from each other, whereas different letters indicate statistical differences (*p* < 0.05).

At every stage of the healing process, macrophages play a crucial role. By releasing various bioactive molecules, macrophages significantly influence the regulation of fibroblast proliferation and function. The presence of macrophages in injured tissue can be quantified using CD68 expression. This study demonstrated that on Day 3, the CD68 expression levels in the other five groups were significantly higher than those in Group M (*p *< 0.05). Additionally, Group D exhibited significantly higher CD68 expression compared to the other five groups (*p *< 0.05). On Days 7 and 14, the CD68 expression levels in the other five groups were markedly lower than those in Group D, with statistically significant differences (*p *< 0.05) (Figure 3). These findings indicate that the LA formulation used in Group D for subcutaneous infiltration anesthesia at the incision site effectively increased the number of macrophages in the injured tissue.

### 3.3 MASSON

**FIGURE 4:**
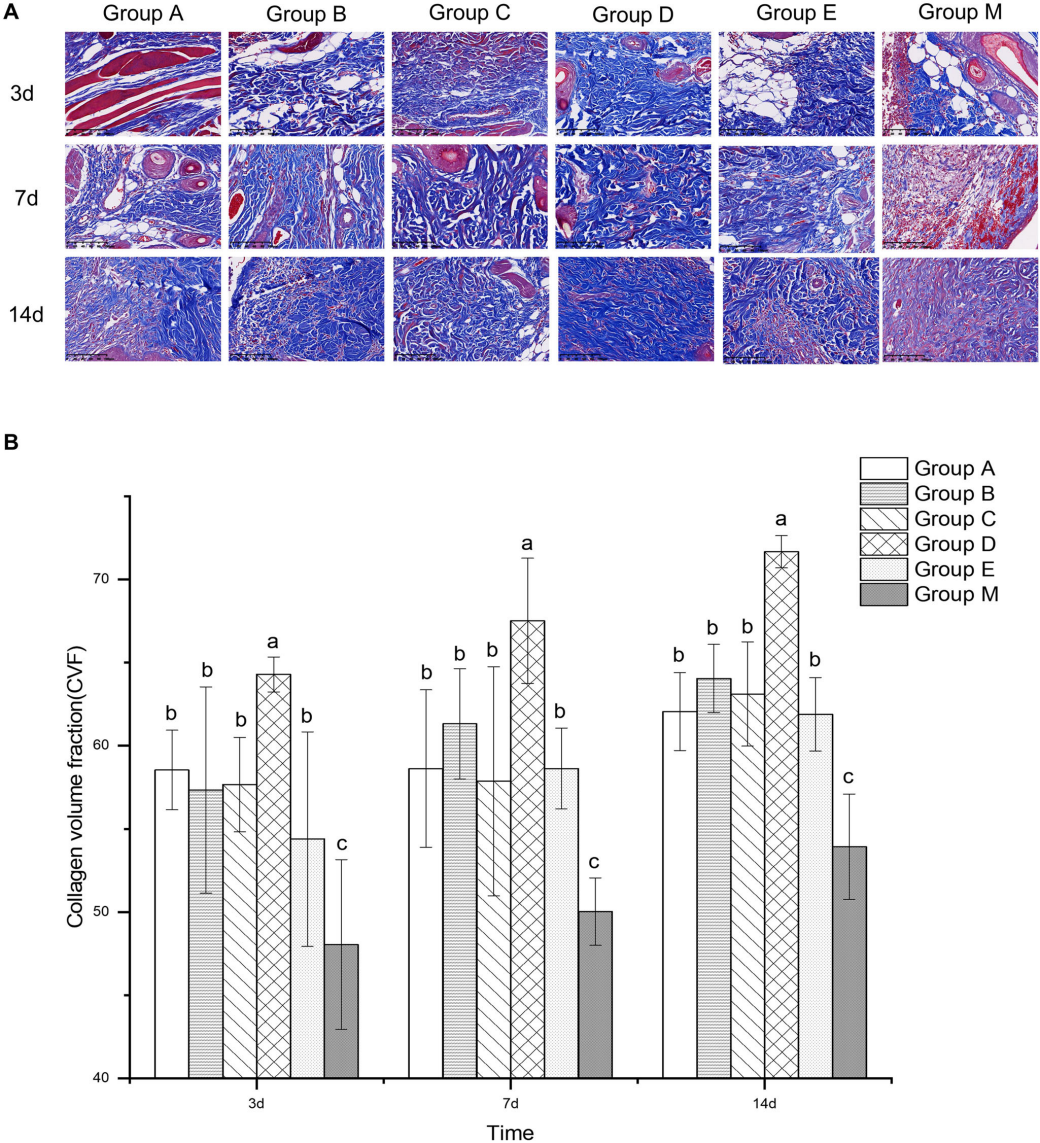
The effects of different anesthetic medication formulations on the level of collagen fiber in the incision tissue on 3, 7, and 14 days after surgery. (A) Representative images showing Masson staining for the level of collagen fiber within incision tissues, at original magnification ×20. (B) Statistical analysis of the CVF in the incision tissue. Data are expressed as means ± SD (*n*  =  6). For comparisons among multiple groups, one‐way ANOVA was utilized. If homogeneity of variance was confirmed, the LSD test was employed for post hoc pairwise comparisons. In cases where the assumption of homogeneity of variance was violated, Tamhane's T2 test was applied. Letters, such as a, b, and c, on bars indicate clustering on the basis of statistical differences. Values labeled with the same letters are not statistically different from each other, whereas different letters indicate statistical differences (*p* < 0.05).

The incision tissues from diabetic rats were stained using the Masson trichrome method, and the CVF was utilized to quantify and compare collagen fiber content. This study aimed to investigate the effects of subcutaneous infiltration anesthesia with ropivacaine combined with various adjuvants on collagen fiber production in incision tissues. The results indicated that on Days 3, 7, and 14 post‐surgery, the CVF values in the five experimental groups were significantly higher than those in Group M (*p *< 0.05). Notably, Group D exhibited a markedly higher CVF compared to the other five groups, with statistically significant differences (*p *< 0.05) (Figure 4). These findings suggest that subcutaneous infiltration anesthesia can promote collagen fiber formation at the incision site, with the formulation used in Group D demonstrating the most pronounced effect.

## 4 Discussion

The body's internal environment undergoes significant alterations during surgery due to multiple external factors, making it susceptible to metabolic stress. This stress can lead to insulin resistance (IR) (S. Zhang, He, et al. 2022), which is particularly harmful to diabetic patients. IR is now understood as a chronic, nonspecific inflammatory process. Severe IR represents a response to traumatic stress that may decrease the tissue's insulin sensitivity index (ISI), disrupt lipid and glucose metabolism, compromise homeostasis, accelerate tissue protein catabolism, induce negative nitrogen balance, markedly reduce the body's resistance to infection and shock, weaken tissue repair capacity, interfere with wound healing, and adversely affect patient prognosis and rehabilitation (Min et al. 2025). The three phases of skin incision healing—inflammation, granulation tissue formation, and remodeling—are initiated immediately upon injury. These stages involve complex and coordinated interactions among various tissues and cells. Extensive cellular and molecular studies have shown the critical roles of numerous cytokines, growth factors, and proteases in the incision‐healing process (Huelsboemer et al. 2024). Otranto et al. (2013) found that IR can impede skin incision healing by prolonging the inflammatory phase, increasing lipid peroxidation levels and TGF‐β expression, disrupting neovascularization, and delaying wound contraction, re‐epithelialization, and granulation tissue formation.

The majority of postoperative pain can be categorized as inflammatory pain. In accordance with the principles of ERAS, an optimal perioperative analgesia protocol ensures a balanced inflammatory response, mitigates surgical stress reactions, prevents excessive inflammation, and promotes rapid patient recovery (Joshi 2023; Karlsen et al. 2024; Schnabel et al. 2024). LIA is a straightforward and effective method that involves the layered administration of long‐acting LAs into the subcutaneous incision site. This technique blocks peripheral nerve sodium ion channels, thereby reducing depolarization and repolarization, prolonging the refractory period, and preventing central sensitization by inhibiting the transmission of nociceptive stimuli from the periphery to the central nervous system (Klasan et al. 2025). LIA is efficacious for various surgical procedures and is characterized by its precise analgesic effect, localized drug action, minimal systemic impact, and low incidence of side effects and complications. It has become a standard practice to administer LAs at the incision site at the conclusion of surgery within the context of multimodal perioperative analgesia (Stamenkovic et al. 2021). Ropivacaine, due to its minimal toxicity to the cardiovascular and central nervous systems, is considered one of the safest LAs and is frequently utilized in postoperative incisional infiltration anesthesia (Maqusood et al. 2024; Sane et al. 2024). While most studies have focused on comparing the analgesic effects of different anesthetic formulations for incisional subcutaneous infiltration, fewer investigations have examined postoperative incision healing. Using a diabetic rat model of postoperative incision injury, this study compared the effects of incisional subcutaneous infiltration anesthesia with ropivacaine combined with various adjuvants on incision healing by modulating the inflammatory response.

The interaction between the insulin signaling pathway and the inflammatory transduction pathway is complex. Inflammatory factors specifically impact certain loci within the insulin signaling pathway, potentially disrupting the signal transduction of insulin receptor substrate (IRS) (Xu et al. 2024). The stress response represents a primary mechanism leading to insulin resistance (IR), with pro‐inflammatory cytokines playing a critical role in both stress response and IR development. Upon binding to its receptor, insulin stimulates receptor autophosphorylation of tyrosine residues, activates kinases, and phosphorylates adaptor proteins such as IRS. Subsequently, IRS can activate phosphatidylinositol 3‐kinase (PI3K) and pyruvate dehydrogenase kinase isozyme 1 (PDK1). This leads to the transduction of signals to protein kinase B (PKB, also known as Akt) and glucose transporter (GLUT), culminating in the translocation of GLUT‐4 vesicles to the plasma membrane to facilitate glucose transport. Disruption at any point along this canonical insulin signaling pathway can impair insulin function, resulting in IR (Bo et al. 2024). The PI3K/Akt signaling pathway, prevalent on the cell surface, plays a pivotal role in transducing signals from membrane receptors into the cell. Extracellular signals such as stress, hypoxia, and ischemia can activate Akt, increasing the expression of phosphorylated Akt (p‐Akt), which promotes cell survival and proliferation while preventing apoptosis (Ran et al. 2023). Based on fundamental experimental investigations, dexmedetomidine exerts its effects through both central and peripheral mechanisms. It inhibits lipid peroxidation and the stress response induced by trauma via the PI3K/Akt signaling pathway. Dexmedetomidine also suppresses the production of pro‐inflammatory cytokines in the immune‐inflammatory response triggered by nociceptive stimuli (C. Ye et al. 2024). Additionally, meta‐analyses have demonstrated that dexmedetomidine, when used as an adjuvant for wound infiltration, significantly enhances the analgesic efficacy of LAs, prolongs the duration of analgesia, reduces pain‐related stress, and prevents postoperative nausea and vomiting (PONV) (Ren et al. 2020). Dezocine, a novel opioid receptor mixed agonist‐antagonist, fully activates κ receptors, partially activates μ receptors without inducing typical μ receptor dependence, and exhibits partial agonist activity at δ receptors. According to Cunha et al., activation of peripheral κ receptors may inhibit inflammatory pain. Moreover, κ receptor agonists can activate the neuronal nitric oxide synthase/nitric oxide (nNOS/NO) signaling pathway via the PI3Kγ/Akt pathway, subsequently activating soluble guanylate cyclase (sGC) to produce cyclic guanosine monophosphate (cGMP), which modulates downstream effectors such as cGMP‐dependent protein kinases and phosphodiesterases (Cunha et al. 2012). In the dorsal root ganglion (DRG) of the spinal cord, this process decreases neuronal excitability, reducing the release of Substance P and other neurotransmitters from sensory nerve endings, thereby inhibiting the transmission of primary nociceptive afferent information. Additionally, it exerts an analgesic effect by attenuating or blocking the transmission of noxious stimuli from afferent neurons to the central nervous system. The study's findings indicated that on Days 3, 7, and 14, incision healing in Group D was significantly superior compared to the other five groups. This suggests that using dezocine and dexmedetomidine as adjuvants to ropivacaine in subcutaneous incisional anesthesia may facilitate wound healing in diabetic rats by mitigating pain stress, regulating inflammatory cytokine expression, preventing insulin resistance (IR), and inhibiting excessive inflammatory responses through the PI3Kγ/Akt signaling pathway.

Reduced angiogenesis, impaired granulation tissue formation, decreased matrix deposition, chronic inflammatory infiltration, enhanced oxidative stress, immunological dysregulation, dysfunction of fibroblasts and keratinocytes, and neuropathy are the primary factors contributing to delayed wound healing in diabetic patients (Q. Huang et al. 2024). To accelerate the healing process of surgical incisions in diabetic patients, promoting the growth of granulation tissue and the regeneration of normal skin adjacent to the incision has emerged as a critical therapeutic target. Granulation tissue plays a pivotal role in wound healing by covering the wound bed and filling the defect, providing temporary scaffolding for revascularization, cell attachment, and tissue development (Y. Huang et al. 2025). Blood vessels, a crucial component of granulation tissue, significantly influence the rate of wound healing (Wallace et al. 2025). CD31, also known as platelet‐endothelial cell adhesion molecule (PECAM‐1), is commonly expressed on the surface of platelets, neutrophils, monocytes, certain T cells, and at the tight junctions between endothelial cells. Immunohistochemical staining with CD31 is frequently used to evaluate vascular development in tissues (Kondo et al. 2022), where positive cells typically form circular or tubular structures. The study results demonstrated that under microscopic examination, Group D exhibited larger vascular tube diameters, more tightly connected vascular endothelial cells, and a favorable neovascular structure conducive to functional recovery. On Day 3, CD31 expression in Group D was significantly higher compared to the other five groups, while Group M showed markedly lower CD31 expression. By Days 7 and 14, CD31 expression in Group D remained notably higher than in the other groups. Dezocine has been shown to reduce serum levels of TNF‐α and IL‐1β, thereby mitigating excessive inflammatory responses and enhancing postoperative recovery (Song et al. 2023). Naltrexone, a pure opioid receptor antagonist that inhibits the opioid growth factor receptor (OGFr), has been shown to promote fibroblast proliferation. It is hypothesized that dezocine may influence fibroblast proliferation and the expression of granulocyte‐macrophage colony‐stimulating factor (GM‐CSF) via the OGF‐OGFr pathway. GM‐CSF, initially identified by Burgess et al. in 1977 through rat lung‐conditioned culture, is a multifunctional hematopoietic factor. By recruiting inflammatory cells, promoting re‐epithelialization, and stimulating angiogenesis, GM‐CSF enhances wound healing (Castro‐Dopico et al. 2020; Martins et al. 2020). Exogenous administration of GM‐CSF has been particularly beneficial for wound healing in patients with diabetes or hydroxyurea‐induced leg ulcers (Fang et al. 2010). According to M. Yan et al. (2018), GM‐CSF treatment at mouse wound sites significantly accelerated wound closure and mitigated microvascular leakage. Additionally, GM‐CSF reduced endothelial permeability by strengthening endothelial junctions and increased Ang‐1 protein production by pericytes. Consequently, GM‐CSF not only initiates the sprouting phase of angiogenesis but also promotes the maturation and stabilization of new microvessels.

In recent years, research has demonstrated that GM‐CSF can promote inflammation, enhance capillary proliferation, and play a crucial role in all stages of wound healing (J. Zhang, Jia, et al. 2022). GM‐CSF accelerates collagen and extracellular matrix synthesis and release by promoting fibroblast migration, proliferation, and mitosis. Moreover, GM‐CSF facilitates the development of granulation tissue and the conversion of actin to fibroblasts, which is dose‐dependent. The findings further confirm GM‐CSF's positive effects on fibroblasts and vascular endothelial cells, as evidenced by significantly higher levels of collagen and hydroxyproline ratios in the GM‐CSF‐treated group compared to the control group. Fibroblasts are essential for synthesizing and secreting collagen fibers, thereby maintaining the function of the extracellular matrix (Rojas et al. 2024). Additionally, low concentrations of ropivacaine administered via subcutaneous infiltration anesthesia at the incision site can promote fibroblast proliferation, upregulate collagen fiber expression, and enhance wound healing. This conclusion aligns with the study results. On Days 3, 7, and 14, Group D exhibited more abundant collagen fiber deposition and significantly higher CVF compared to the other five groups. In contrast, Group M had less collagen fiber deposition and much lower CVF. Furthermore, the collagen fibers in Group D were more densely arranged, effectively preventing excessive collagen fiber proliferation and scar tissue formation. Effective wound contraction, facilitated by the deposition and orderly arrangement of collagen fibers, is beneficial for shortening healing time and enhancing wound tissue remodeling (Gardeazabal and Izeta 2024; Zeng et al. 2019). According to the study by Lazarus 1972, excessive collagen deposition at the site of injury can disrupt normal tissue architecture, while insufficient collagen deposition may result in suboptimal tissue repair. The most common consequences include weakened wound tissue and inadequate tensile strength.

We hypothesized that dezocine would enhance the expression of GM‐CSF through the OGF‐OGFr pathway, thereby promoting macrophage recruitment to the incision site and stimulating the secretion of significant amounts of EGF, FGF, PDGF, and other biochemical factors. Dezocine accelerated the inflammatory response by facilitating the clearance of germs, foreign bodies, and cellular debris from the wound's periphery via lysosomal peroxide activity. The study's findings revealed that on Day 3, Group D exhibited markedly higher CD68 expression compared to the other five groups. Conversely, Group M showed significantly lower CD68 expression relative to the other five groups. On Days 7 and 14, Group D maintained notably higher CD68 expression levels compared to the other groups. Group D contained dexmedetomidine, an α2 receptor agonist, which exerts anti‐inflammatory effects by suppressing inflammatory mediators and inhibiting sympathetic nerve excitability, in addition to its sedative and analgesic properties (Nawan et al. 2025). Currently, dexmedetomidine is predominantly administered intravenously; however, local administration has been reported to have a more rapid onset of action, preventing drug redistribution and improving therapeutic outcomes (Pereira et al. 2025). A previous study evaluated ropivacaine both as a standalone agent and in combination with dexmedetomidine, demonstrating a substantial anti‐inflammatory effect when used in conjunction (J. Li et al. 2017). Dexmedetomidine not only inhibits the release of pro‐inflammatory cytokines (e.g., IL‐6, TNF‐α, IL‐8, and IL‐1β) but also prevents neutrophil and macrophage migration to the inflammation site. Additionally, it enhances the concentration of anti‐inflammatory cytokines, exhibiting comparable anti‐inflammatory efficacy to methylprednisolone (Can et al. 2009; L.‐C. Li et al. 2021; Strada et al. 2024). Incision subcutaneous infiltration anesthesia using dezocine and dexmedetomidine as adjuvants to ropivacaine, according to this study, can enhance CD68 expression around the incision site, promote macrophage accumulation in the periwound area, mitigate excessive inflammation, preserve a healthy inflammatory response, and facilitate wound healing.

This study identifies several limitations and future prospects. First, the findings indicate that the use of dezocine and dexmedetomidine as adjuvants to ropivacaine in subcutaneous infiltration anesthesia significantly enhances incision healing in diabetic rats. However, the precise mechanisms or pathways responsible for this effect remain to be elucidated through further research. Second, it is important to note that this study was conducted as an animal experiment. To validate the therapeutic efficacy of this regimen, additional clinical trials are necessary. This approach may offer novel strategies for postoperative multimodal analgesia and introduce innovative concepts for managing postoperative pain in diabetic patients.

## 5 Conclusion

In conclusion, the adjunctive use of dezocine and dexmedetomidine with ropivacaine in incisional subcutaneous infiltration anesthesia may effectively mitigate pain‐related stress, modulate the expression of inflammatory cytokines, prevent ischemia‐reperfusion (IR) injury, inhibit excessive inflammation while preserving beneficial inflammatory responses, upregulate GM‐CSF expression, promote fibroblast proliferation, stimulate capillary growth, enhance collagen fiber deposition, and facilitate macrophage aggregation at the wound edge via the PI3Kγ/Akt signaling pathway and OGF‐OGFr axis. These mechanisms collectively contribute to improved wound healing.

## Author Contributions

**Lang Yu**: data curation, formal analysis, investigation, writing – original draft, writing – review and editing. **Bin Gao**: data curation, formal analysis, writing – review and editing. **Lingling Sun**: data curation, formal analysis, writing – review and editing. **Jing Mu**: investigation, methodology, software, writing–review and editing. **Piaopiao Zhang**: investigation. **Qin Zhang**: investigation. **Huanzhong He**: funding acquisition, writing – review and editing, supervision. **He Liu**: writing – review and editing, supervision.

## Funding

This work was supported by the Scientific Research Funding of Huzhou Science and Technology Bureau, awarded to Dr. Huanzhong He (Project number: 2021GZB11).

## Conflicts of Interest

The authors declare no conflicts of interest.

## Data Availability

The data that support the findings of this study are available from the corresponding author upon reasonable request.
